# Sequential Inoculation of Native Non-*Saccharomyces* and *Saccharomyces cerevisiae* Strains for Wine Making

**DOI:** 10.3389/fmicb.2017.01293

**Published:** 2017-07-18

**Authors:** Beatriz Padilla, Laura Zulian, Àngela Ferreres, Rosa Pastor, Braulio Esteve-Zarzoso, Gemma Beltran, Albert Mas

**Affiliations:** Departament de Bioquímica i Biotecnologia, Facultat d’Enologia, Universitat Rovira i Virgili Tarragona, Spain

**Keywords:** indigenous yeast, *Torulaspora*, *Metschnikowia*, *Hanseniaspora*, *Starmerella*, Priorat, wine

## Abstract

The use of non-*Saccharomyces* yeast for wine making is becoming a common trend in many innovative wineries. The application is normally aimed at increasing aromas, glycerol, reducing acidity, and other improvements. This manuscript focuses on the reproduction of the native microbiota from the vineyard in the inoculum. Thus, native selected yeasts (*Hanseniaspora uvarum, Metschnikowia pulcherrima, Torulaspora delbrueckii, Starmerella bacillaris* species and three different strains of *Saccharomyces cerevisiae*) were inoculated sequentially, or only *S. cerevisiae* (three native strains together or one commercial) was used. Inoculations were performed both in laboratory conditions with synthetic must (400 mL) as well as in industrial conditions (2000 kg of grapes) in red winemaking in two different varieties, Grenache and Carignan. The results showed that all the inoculated *S. cerevisiae* strains were found at the end of the vinifications, and when non-*Saccharomyces* yeasts were inoculated, they were found in appreciable populations at mid-fermentation. The final wines produced could be clearly differentiated by sensory analysis and were of similar quality, in terms of sensory analysis panelists’ appreciation.

## Introduction

Due to the increasingly competitive global market, there is a trend for local wine producers to attempt to link their products with geographical identity (Harvey et al., 2014), which has been identified as the *terroir*, including soil, climate, grape varieties and microbial population (Bokulich et al., 2013). Native microorganisms, particularly yeasts, have been highlighted as key factors for preserving typicality, characteristic flavors and the high quality of wines (Tofalo et al., 2014), which could be considered the microbial fingerprint. However, this microbial fingerprint is not probably static and can change along the time and climatic conditions of the harvest as can be seen by comparing in the Priorat region results form our group (Torija et al., 2001; Padilla et al., 2016). Also new results obtained in a 3 years study (Vigentini et al., 2015) are opening the debate if the microbial population permanently remains in vineyards.

The Priorat Qualified Appellation of Origin (DOQ in Catalan language) is a traditional area for wine production located in the south of Catalonia, Spain, where Carignan and Grenache are the typical and characteristic red grape varieties. Although limited data are available concerning the use of locally selected yeast for must inoculation in Catalonia, several studies developed in different wine-producing areas have noted the use of native yeasts as an innovative approach to obtain wines reflecting *terroir* (Vilanova and Massneuf-Pomarède, 2005; Carrascosa et al., 2012; Scacco et al., 2012).

The use of locally selected yeast is normally based on a study on natural biodiversity. Yeast biodiversity during the spontaneous fermentation of grape juice includes the presence of different species. It has been widely reported that non-*Saccharomyces* species dominate the first phase of alcoholic fermentation, and some of these yeasts can also be present at advanced stages, even while the species *Saccharomyces cerevisiae* dominates the process (Fleet, 1993). This extensive yeast biodiversity is the reason supporting the design and implementation of yeast starter cultures that are not pure or single-species. The defense of the wine typicality should actually include a combination of non-*Saccharomyces* and *S. cerevisiae* strains with the aim to obtain wines exhibiting complexity but avoiding the risks related to natural fermentations (Comitini et al., 2011; Tristezza et al., 2011; Suzzi et al., 2012; Gobbi et al., 2013; Medina et al., 2013).

Thus, the proper design of an autochthonous starter culture is essential to reproduce the local sensory properties, including the incorporation of a mixture of different non-*Saccharomyces* species and different strains of *S. cerevisiae* to mimic spontaneous alcoholic fermentations. Among non-*Saccharomyces* species, *Hanseniaspora uvarum*, *Starmerella bacillaris* (previously known as *Candida zemplinina*), *Torulaspora delbrueckii* and *Metschnikowia pulcherrima* have been isolated in different wines (Lopandic et al., 2008; Kraková et al., 2012; Albertin et al., 2014) and have been described as characteristic of the Priorat (Torija et al., 2001; Wang et al., 2015; Padilla et al., 2016; Portillo and Mas, 2016). However, the combination of several non-*Saccharomyces* and strains of *S. cerevisiae* can be challenged by the winemaking conditions (i.e., SO_2_ dosage, temperature, etc…) as well as the initial yeast population in grapes (Constantí et al., 1998; Vigentini et al., 2014). Thus, special care in the winery has to be taken for this kind of procedures.

This work aims to test the industrial use of locally selected yeast strains reproducing the vineyard for wine production in the Priorat DOQ. For this purpose, a specific multistarter culture consisting of different strains of *S. cerevisiae* and non-*Saccharomyces* species mimicking Priorat natural musts has been developed. This study was done using synthetic must in order to have all the conditions of incubation and sterility under control as well as natural Grenache and Carignan grape juices at industrial scale. The mix of different species was used to inoculate the four non-*Saccharomyces* species and sequentially (24 h later) the mix of three different *S. cerevisiae* strains. Additionally, control fermentations containing only the three native *S. cerevisiae* strains or a *S. cerevisiae* commercial strain have been performed to evaluate the contribution of non-*Saccharomyces* and native inoculum to fermentation kinetics, yeast dynamics, and different oenological parameters as well as the production of major volatile compounds. Additionally, a sensory evaluation based on triangle tests was performed.

## Materials and Methods

### Strains

Four non-*Saccharomyces* yeast strains: *H. uvarum* CECT 13130, *S. bacillaris* CECT 13129, *T. delbrueckii* CECT 13135 and *M. pulcherrima* CECT 13131; and three *S. cerevisiae* strains: CECT 13132, CECT 13133 and CECT 13134, were used in this work. All strains were previously isolated from DOQ Priorat spontaneous fermentations (Padilla et al., 2016) and deposited in the Spanish Type Culture Collection (CECT). The non-*Saccharomyces* species were selected by the absence of off-odor production (especially acetic acid), prevalence in musts during fermentations and ester production. Instead, resistance to high sugar concentration was the main criteria for selection of *S. cerevisiae* strains, which is one of the main characteristics of Priorat musts, but also competitiveness in front of other *Saccharomyces* strains and the production of esters and acetates in single fermentations (Torija et al., 2001). Additionally, commercial *S. cerevisiae* wine strains GR in Grenache (provided by AB Mauri, Sydney, NSW, Australia), CA in Carignan or QA23 in Synthetic must (both from Lallemand Inc., Montreal, QC, Canada) were included in this study as a control. Yeasts were maintained in glycerol stocks at -80°C.

### Biomass Production

Native yeasts were grown in plates with 25 cm of diameter containing YPD medium (10 g/L yeast extract, 20 g/L peptone, 20 g/L glucose, 17 g/L agar) at 28°C before use. Plates were washed with saline solution (NaCl 0.9% w/v) for yeast collecting, and cells were then quantified and used as inocula for laboratory and industrial vinifications. Commercial *S. cerevisiae* strains were purchased as active dry yeast and rehydrated following the manufacturer’s instructions.

### Fermentation and Sampling

The laboratory scale fermentations were conduct using synthetic must (as reported in Andorrà et al., 2012) with nitrogen content of 300 mg/L and 200 g/L of total sugar and pH adjusted to 3.3. Fermentations were performed in triplicate in continuous shaking at 120 rpm at 25°C in 500 mL glass bottles filled each one with 400 mL of synthetic must and covered with cotton caps. The inoculation process is described in **Table 1**, monitoring and sampling was done as in the industrial scale.

**Table 1:** Yeast composition of starter cultures employed in different fermentation modalities (cells/mL).

	Grenache			Carignan and Synthetic must		
		
Yeast strains	A	B	C	A	B	C
*H. uvarum* CECT 13130	1.2×10^5^			1.2×10^6^		
*S. bacillaris* CECT 13129	6×10^4^			6×10^5^		
*T. delbruecki* CECT 13135	10^4^			10^5^		
*M. pulcherrima* CECT 13131	10^4^			10^5^		
*S. cerevisiae* CECT 13132	7×10^4^	7×10^4^		7×10^5^	7×10^5^	
*S. cerevisiae* CECT 13133	7×10^4^	7×10^4^		7×10^5^	7×10^5^	
*S. cerevisiae* CECT 13134	7×10^4^	7×10^4^		7×10^5^	7×10^5^	
*S. cerevisiae* GR			2×10^6^			
*S. cerevisiae* CA						2×10^6^
*S. cerevisiae QA 23*						2×10^6^


On the other hand, six industrial fermentations were conducted in stainless steel tanks filled with 2000 kg of crushed grapes, rendering 1050 L of Grenache (GR) or Carignan (CA) wine in a cellar from DOQ Priorat. Due to the specific characteristics of the vineyards in Priorat, this volume is very common in the area and is the volume routinely used in this cellar. Before inoculation, musts were chemically analyzed. The musts had a density around 1100 g/L, pH between 3.19 and 3.29 with total acidity of 4.6 and 5.2 g/L, which are typical values from the area. Due to the low levels of yeast assimilable nitrogen (66 and 80 mg/L), juices were gradually supplemented throughout the alcoholic fermentation with a total of 50 mg inorganic nitrogen/L (as Diammonium Phosphate) and 15 mg organic nitrogen/L (as yeast lysates). For each must variety, three vinifications containing different yeast strain combinations were performed and monitored (**Table 1**). The A fermentations (A-SM = in Synthetic must, A-GR = in Grenache must and A-CA = in Carignan must) contained a combination of the seven native strains, which were sequentially inoculated. At time 0, non-*Saccharomyces* strains were added into the must, mimicking the natural yeast composition found in previous studies, and the mixture of *S. cerevisiae* was incorporated 24 h later. In contrast, the B fermentations (B-SM, B-GR and B-CA) contained only the mixture of the three *S. cerevisiae* autochthonous strains. Experiments with commercial *S. cerevisiae* strains (C-SM, C-GR and C-CA) were conducted as a control for each type of must.

From each bottle and tank, daily samples were taken to monitor sugar concentration by measuring must density using an electronic densitometer (Mettler-Toledo S.A.E., Barcelona, Spain). Additionally, samples of the grape juice before inoculation (day 0), 1 day after inoculation with non-*Saccharomyces* in the case of mixed fermentations (24 h; day 1), 1 day after inoculation with *S. cerevisiae* (24 h Sc; day 1 or 2), at a mid-fermentation point (M; day 4–5) and at the end of the fermentation (F; day 8–9) were also aseptically withdrawn for yeast counting and molecular identification. Moreover during industrial fermentations, cells from 1 mL at each sampling point were collected after centrifugation (Spectrafuge, Labnet, United States) at 9200 g for 5 min for quantitative PCR (qPCR) analysis.

The synthetic wines were analyzed after the alcoholic fermentation. The final industrial wines were stabilized for 30 days at 4°C, and then 30 ppm of sulfur dioxide was added as potassium metabisulfite, and the final product was bottled. These conditions were maintained for 2 months until the sensory evaluation took place.

### Yeast Content and Isolation

Yeast counts were conducted in duplicate on solid YPD and agar-Lysine (LYS) plates (Oxoid, United Kingdom, prepared according to manufacturer’s instructions) after serial decimal dilution with distilled sterile water of the samples. Plates were incubated at 28°C for 3 days. For yeast isolation and identification, from plates containing 30–300 colonies approximately, 25 colonies from each medium and each sampling point were picked randomly.

### Yeast Identification: RFLPs of 5.8S-ITS rRNA Region and Sequencing of D1/D2 of 26S rRNA Gene

Yeast isolates were identified by PCR-RFLP analysis of 5.8S-ITS rDNA according to Esteve-Zarzoso et al. (1999), using primers ITS1 and ITS4 (White et al., 1990). PCR products were digested, without further purification, with the restriction enzymes *Cfo*I, *Hae*III, *Dde*I, and *Hin*fI. The PCR products and their restriction fragments were separated by gel electrophoresis on 1.5 and 3% (w/v) agarose gels, respectively. The sizes of the DNA fragments were estimated by comparison against a DNA ladder (100 bp Roche Diagnostics GmBh, Mannheim, Germany). The obtained restriction profiles were compared with the profiles reported in Esteve-Zarzoso et al. (1999) and in the Yeast-id database^1^.

Sequencing of the D1/D2 domains of 26S rRNA gene was conducted to confirm yeast identification using primers NL1 and NL4 (Kurtzman and Robnett, 1998). The PCR product was purified and sequenced by Macrogen Inc. (Seoul, South Korea) using an ABI3730XL automated capillary DNA sequencer. The sequences were compared with the ones in GenBank using the BLASTN tool (NCBI) and deposited in GenBank database with the accession numbers described in Padilla et al. (2016).

### Yeast Typing

The isolates from the dominant yeast species were genetically characterized. *S. cerevisiae* strains were typified by the analysis of inter-delta regions, as described by Legras and Karst (2003) using the primers delta12 and delta21. DNA was extracted from yeast cultures grown in YPD broth for 24 h at 28°C (Querol et al., 1992). Interdelta PCR products were separated by electrophoresis on 2% (w/v) agarose gels. The sizes of the DNA fragments were estimated by comparison against a DNA ladder (100 bp Roche Diagnostics GmBh, Mannheim, Germany).

### Quantitative PCR

Yeast DNA was extracted from 1 ml pelleted cells using the DNeasy PLANT kit (Qiagen, United States). Quantitative PCR (qPCR) was performed in a 7300 Fast Real-Time PCR System (Applied Biosystems, Foster City, CA, United States). Power SybrGreen master mix was used according to the manufacturer’s instructions (Applied Biosystems, Foster City, CA, United States). An AB 0–600 96-well optical plate (Thermo Scientific, Waltham, MA, United States) was used for the reaction. The primers used for each species were those described by Hierro et al. (2007) (*Saccharomyces* and *Hanseniaspora*), Andorrà et al. (2010) (*S. bacillaris*), Zott et al. (2010) (*T. delbrueckii*), and Díaz et al. (2013) (*M. pulcherrima*). The cycle threshold (CT) was automatically determined. All samples were analyzed in duplicate, and cell concentrations were quantified by CT measurement using the calibration curves of a relevant concentration series of yeast cells for each species (see calibration curves for each species in Supplementary Table S1).

### Chemical Analysis of Musts and Wines

Density, pH, total acidity and ethanol were determined according to the methods in the Compendium of International Methods of Analysis of Musts and Wines (OIV, 2015). Yeast assimilable nitrogen was measured according to the formol method (Gump et al., 2000). Sugars (glucose and fructose), acetic acid and glycerol were quantified using the Miura one enzymatic autoanalyzer (BioGamma I.S.E. S.r.L., Rome, Italy) with the corresponding enzymatic kits (BioSystems S.A., Barcelona, Spain).

### Determination of Volatile Compound Production

The six final wines obtained using industrial conditions were analyzed for major volatile compounds by gas chromatographic–flame ionization detection (GC-FID) by an external analytical service (L.A.A.E., Zaragoza, Spain) according to Ortega et al. (2001). In summary, 3 mL of each wine were diluted with 7 mL of water, salted with 4.5 g of ammonium sulfate and extracted with 0.2 mL of dichloromethane. The extract was injected in the split mode into a Varian CP-3800 GC system (Palo Alto, CA, United States), separated on a DB-WAX polyethylene glycol column (30 m × 0.32 mm and 0.5 μm film thickness) from J&W Scientific (Folsom, CA, United States), and detected by FID.

### Sensory Analysis of the Industrial Wines Obtained

The panel for wine sensory evaluation consisted of two groups of tasters. Group A consisted of eleven judges (six females and five males) recruited from the Faculty of Oenology of the University Rovira i Virgili. Group B consisted of six oenologists from cellars belonging to the Priorat DOQ (four females and two males). Panelists were placed in individual sensory booths. Fifty milliliters of each wine was served at room temperature, and the order of presentation was randomized. For each grape variety, two different discriminating triangle tests were presented, one containing samples from fermentations A and B and the other from B and C.

### Statistical Analysis

Significant differences in sensory analysis were defined using the critical number of correct answers for the triangle test (Roessler et al., 1948).

## Results

### Yeasts in Natural Musts

A specific characteristic of these juices was the very healthy status of the grapes, which reached concentrations of 2 × 10^3^ (GR) to 4 × 10^4^ (CA) CFU/mL when plated. Yeast populations in the grapes of the area are generally higher, approximately 10^5^ cells/g grapes or mL of must. A total of 153 colonies were identified from these musts. This low yeast population, especially in GR juices, led to the isolation of many different yeast species, the most abundant being *Rhodotorula mucilaginosa* (30%), *Debaryomyces hansenii* (21%), and *M. pulcherrima* (19%). Additionally, other species such as *Wickerhamomyces anomalus* and *Zygoascus hellenicus* were isolated in minor numbers (less than 10%). Instead, in the case of CA musts, the more common *H. uvarum* (74%) and *S. bacillaris* (25%) were present, reaching 99% of the isolates, and only one additional isolate of *M. pulcherrima* was found.

### Fermentation Kinetics and Yeast Population

**FIGURE 1:**
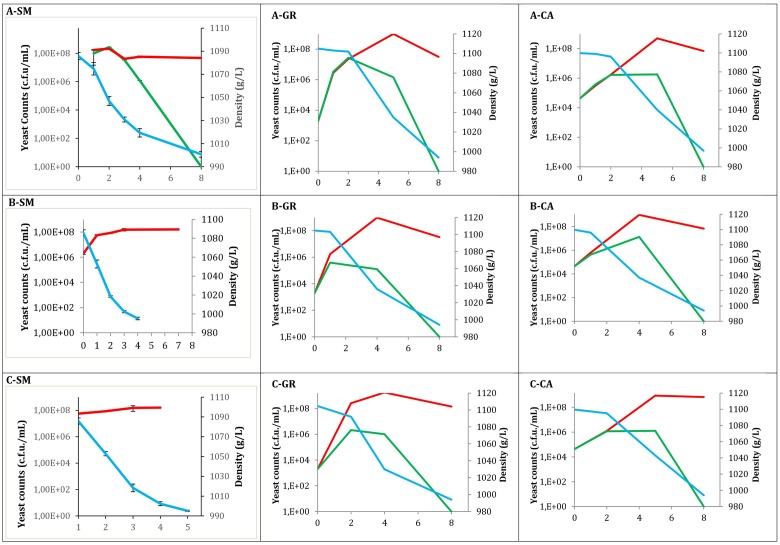
Evolution of different fermentations modalities. Total yeast (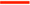) counts obtained from YPD; Non-*Saccharomyces* yeast (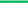) counts on LYS; and Density (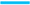). **(A)** Mixed fermentation (four species of non-*Saccharomyces* and the three strains of *Saccharomyces*), **(B)** Fermentation performed using the three native strains of *Saccharomyces*, **(C)** Fermentations conducted by industrial yeast starter belonging to *Saccharomyces*. Fermentations were performed using different musts. SM, Synthetic must; GR, Grenache must; CA, Carignan must.

Total yeast counts (YPD), non-*Saccharomyces* yeast counts (LYS) and must density throughout all fermentations are shown in **Figure 1**. Values at time 0 correspond to must samples before inoculation in the case of industrial fermentations, while for synthetic must fermentations correspond to the inoculated population. In all cases, the typical growth kinetic was observed, exhibiting high total yeast viability until the end of the fermentations, with values of approximately 10^8^ CFU/mL. In contrast, there was no growth of non-*Saccharomyces* species at this point, with counts at the middle fermentation point ranging between 10^5^ and 10^7^ CFU/mL. When non-*Saccharomyces* yeasts were inoculated (**Figure 1A**), the population recovered in LYS plates reached concentrations of 10^6^ (A-CA) to 10^8^ (A-SM) CFU/mL. These non-*Saccharomyces* populations decreased when *Saccharomyces* was inoculated to synthetic must fermentations; however, in natural musts this high population size was maintained until the middle of fermentation, declining afterward. Additionally, three strains of *S. cerevisiae* were sequentially inoculated after 24 h. When the three *S. cerevisiae* native strains were inoculated (**Figure 1A**), according to the type of must used, a similar pattern was observed. In all fermentations the non-*Saccharomyces* population was able to increase during the 1st days to decrease afterward. However, in synthetic must, the decreased appears shortly after *S. cerevisiae* inoculation, whereas in natural musts these decreases were later.

Mixed fermentation in synthetic must revealed the maximum yeast diversity on YPD plates at mid fermentation. During the initial sampling points, a high presence of *H. uvarum* was detected; however, these non-*Saccharomyces* yeast species were not identified at the last sampling point, in which all of the colonies were identified as *S. cerevisiae*.

When the three selected *S. cerevisiae* strains were used (**Figure 1B**) the non-*Saccharomyces* populations had a similar pattern, with Grenache must samples reaching slightly lower populations. When the commercial yeast strains were used (**Figure 1C**) the pattern was also very similar to the inoculation of the three *S. cerevisiae* strains.

The fermentation kinetics observed by density monitoring showed that the fermentations finished within 8 days when a mixture of *Saccharomyces* and non-*Saccharomyces* are present, independently of the origin (natural or synthetic) of the must. However, for fermentation using *Saccharomyces* inoculation this time is reduced to 4 days (synthetic must). This fact can be explained for the presence of lag phase when non-*Saccharomyces* yeasts are present. In the case of GR mixed fermentation, the sugar consumption was slightly slower compared with pure *S. cerevisiae*, although the process finished at the same time. For the three CA experiments, a similar fermentative pattern was observed.

### Yeast Population Dynamics

**FIGURE 2:**
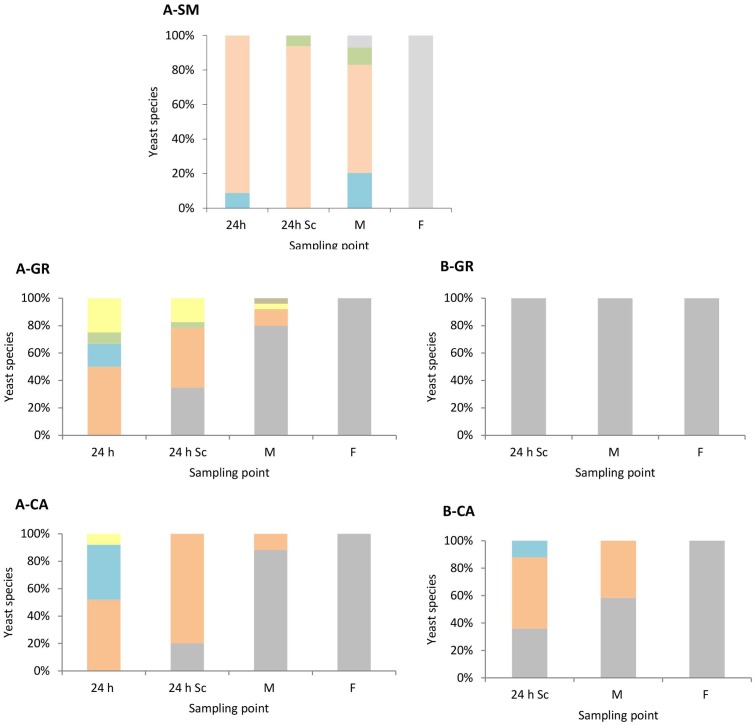
Yeast species population dynamics established by RFLP-ITS-PCR of YPD-cultured isolates. **(A)** Mixed fermentation (four species of non-*Saccharomyces* and the three strains of *Saccharomyces*), **(B)** Fermentation performed using the three native strains of *Saccharomyces*. SM, Synthetic must; GR, Grenache must; CA, Carignan must. The species identified were *Hanseniaspora guilliermondii* 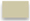, *Metschnikowia pulcherrima* 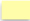, *Torulaspora delbrueckii* 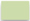, *Starmerella bacillaris* 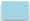, *Hanseniaspora uvarum* 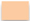, *Saccharomyces cerevisiae* 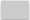. Columns are indicated as: 24 h (24 h after non-*Saccharomyces* inoculation), 24 h Sc (24 h after *S. cerevisiae* inoculation), M (middle fermentation), F (end of fermentation).

As expected, the colonies recovered on YPD medium (**Figure 2**), at later time sampling points were identified as *S. cerevisiae* (100%). Only *S. cerevisiae* was recovered from the colonies isolated in synthetic must fermentations inoculated with this species (data not shown). However, in natural must fermentations, a clear difference was observed between mixed (**Figure 2A**) and pure *S. cerevisiae* vinifications (**Figure 2B**). In the natural must fermentations inoculated with a mixture of non-*Saccharomyces*, a higher biodiversity during the first stages of the process was observed compared with those inoculated with *S. cerevisiae*. In the case of A-GR, all inoculated non-*Saccharomyces* species, *H. uvarum* (50%), *S. bacillaris* (17%), *T. delbrueckii* (8%), and *M. pulcherrima* (25%), were recovered after 1 day of inoculation (24 h); however, in A-CA, all inoculated yeasts except *T. delbrueckii* were found, with values of 52% for *H. uvarum*, 40% for *S. bacillaris* and 8% for *M. pulcherrima*. Additionally, *H. guilliermondii* was also recovered at the middle point in A-GR. This high biodiversity present in mixed inocula fermentations decreased as the fermentation proceeded. The percentages of non-*Saccharomyces* species found were between 65 and 80% 24 h after inoculation with *S. cerevisiae* and 12 and 20% at the mid-fermentation point.

Once *S. cerevisiae* was incorporated into the must, it was possible to isolate it after 24 h (24 h Sc). In A-GR as well as in A-CA, *S. cerevisiae* gradually dominated the process. In the fermentation with the native strains of *S. cerevisiae* B-GR, only the yeast *S. cerevisiae* was isolated at the three sampling points. In contrast, in B-CA, the imposition occurred gradually, as non-*Saccharomyces* species were also recovered up to the mid-fermentation point (42%).

**FIGURE 3:**
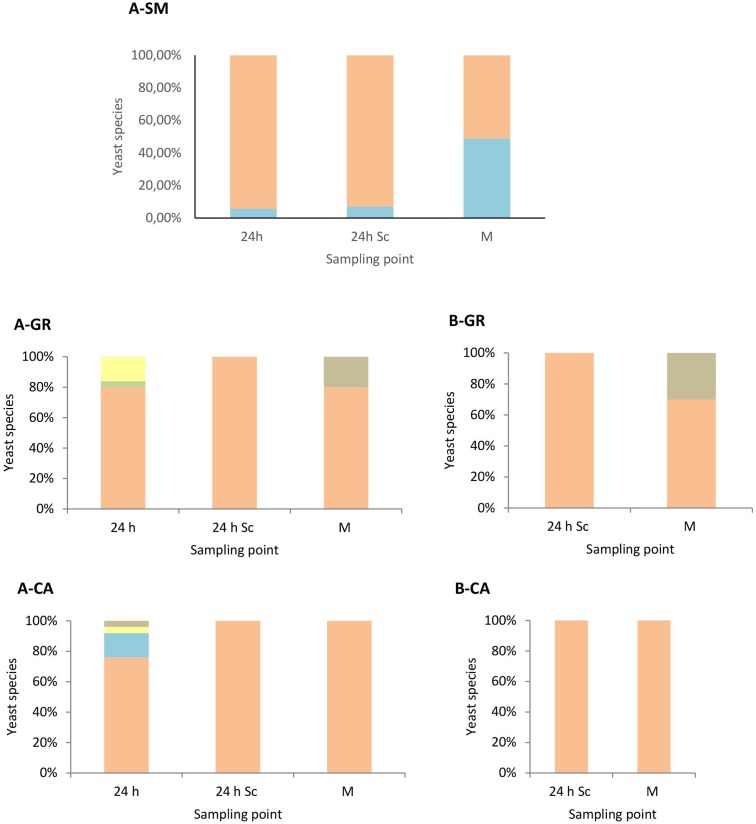
Yeast species population dynamics established by RFLP-ITS-PCR of LYS-cultured isolates. **(A)** Mixed fermentation (four species of non-*Saccharomyces* and the three strains of *Saccharomyces*), **(B)** Fermentation performed using the three native strains of *Saccharomyces*. SM, Synthetic must; GR, Grenache must; CA, Carignan must. The species identified were *Hanseniaspora guilliermondii* 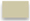, *Metschnikowia pulcherrima* 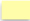, *Torulaspora delbrueckii* 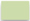, *Starmerella bacillaris* 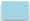, *Hanseniaspora uvarum* 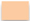, *Saccharomyces cerevisiae* 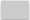. Columns are indicated as: 24 h (24 h after non-*Saccharomyces* inoculation), 24 h Sc (24 h after *S. cerevisiae* inoculation), M (middle fermentation), F (end of fermentation).

These results were confirmed when the yeast population dynamics in LYS media were analyzed, where non-*Saccharomyces* species were isolated until the mid-fermentation point but undetectable at later stages (**Figure 3**). In synthetic must fermentations only *H. uvarum* and *S. bacillaris* were detected, showing an increase of the *S. bacillaris* presence as the fermentation proceeded (**Figure 3A**). In natural must fermentations inoculated with non-*Saccharomyces* yeast (**Figure 3A**) showed some species diversity at the beginning of the fermentation compared with pure *S. cerevisiae* fermentations (**Figure 3B**), although the most abundant yeast in all cases was *H. uvarum*, with proportions of approximately 86% and 95% in the GR and CA varieties, respectively. Among non-*Saccharomyces* species, this yeast dominated throughout fermentation, with just one non-inoculated species, *H. guilliermondii*, detected in small percentages at mid-fermentation in the case of GR wines.

**FIGURE 4:**
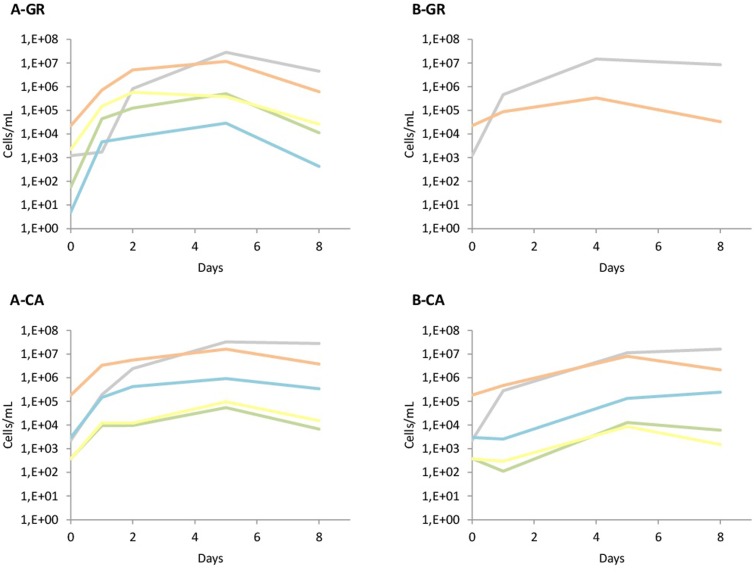
Population dynamics of different yeast species established by qPCR. The species tested were Saccharomyces spp. (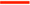); *Hanseniaspora* spp. (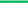); *S. bacillaris* (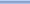); *T. delbrueckii* (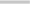); *M. pulcherrima* (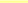). **(A)** Mixed fermentation (four species of non-*Saccharomyces* and the three strains of *Saccharomyces*), **(B)** Fermentation performed using the three native strains of *Saccharomyces*. GR, Grenache must, CA, Carignan must.

The yeast dynamics for natural must fermentations were also analyzed by culture-independent techniques, specifically by qPCR (**Figure 4**). The data obtained from qPCR analysis overall agree with the plating results, with some particularities. First, the quantification of yeast in both musts in GR showed the presence of *Hanseniaspora species* at higher levels (1 × 10^4^ cells/mL) than the colonies recovered on plates (2 × 10^3^ CFU/mL). Furthermore, *S. cerevisiae* initial counts detected by qPCR were at concentrations that had to be detected in plates, although no *S. cerevisiae* isolates were identified using the RFLPs of 5.8S ITS rDNA. However, when *S. bacillaris* was recovered on plates, its quantification by qPCR was very low. In contrast, in the CA musts, the massive presence of *Hanseniaspora* cells agreed with the observations on plates, as well as the numbers of *S. bacillaris*, although in this case the qPCR counts were slightly lower than expected.

The increase of cell concentration due to inoculation was observable in all cases when the addition of the starter culture was performed. When the non-*Saccharomyces* species were inoculated, the observed increase agreed with the inoculated populations, except for *S. bacillaris* in GR, likely due to its low presence in the grape juice (**Figure 4A**). In the musts inoculated with autochthonous *S. cerevisiae* (**Figure 4B**), the increase was also seen in the quantification with qPCR. Finally, the levels of non-*Saccharomyces* during all these fermentations were very similar to the levels detected on LYS plates, with a clear dominance of *H. uvarum*. The presence of non-*Saccharomyces* throughout B-CA fermentation is remarkable, likely due to the presence of higher populations of these yeasts in the CA must.

### Yeast Typing

To test the dominance of the major inoculated species, *S. cerevisiae* strains isolated during the fermentations were typified at strain level. In the case of *S. cerevisiae*, the analysis was performed using the colonies at the end of fermentations. In fermentations A and B, the interdelta fingerprint of *S. cerevisiae* colonies isolated at the end of the process corresponded with the three native strains inoculated, CECT 13132, CECT 13133 and CECT 13134. Although the three profiles were present at the end of the fermentations, the main profile recovered was that of the strain CECT 12132, followed by CECT 12134 and CECT 12133 (**Table 2**). In the fermentations with the commercial strain, only the inoculated strain was recovered (data not shown).

**Table 2:** Percentages of the inoculated *S. cerevisiae* strains recovered at the end of different fermentations.

	CECT 13132	CECT 13133	CECT 13134
A-SM	54	18	27
B-SM	56	25	18
A-GR	48	8	44
B-GR	63	8	29
A-CA	68	14	18
B-CA	64	12	24


*A, Mixed fermentation (four species of non-*Saccharomyces* and the three strains of *Saccharomyces*); B, Fermentation performed using the three native strains of *Saccharomyces*. Fermentations were performed using different musts: SM, Synthetic must; GR, Grenache; CA, Carignan.*

### Chemical Analysis of Wines

The main oenological parameters of the wines obtained are shown in **Table 3**. All wines contained less than 2 g/L of residual sugars. Additionally, all wines presented an alcohol level expected according to the sugar content of the musts. Small variations were observed in the synthetic and CA wines, with alcohol content from 12.43 to 12.83% for synthetic must or from 13.9 to 14.3% for Carignan wines. Within each wine, the natural must fermentations performed with commercial *S. cerevisiae* strains contained higher levels of glycerol and acetic acid than fermentations performed with autochthonous strains, however, when synthetic must was used, the highest values were exhibited by the mixed fermentation (**Table 3**).

**Table 3:** Analytical parameters of final wines.

	Glucose+Fructose (g/L)	Glycerol (g/L)	Acetic acid (g/L)	Alcohol (% v/v)	pH
A-SM	3.79 ± 1.50	11.06 ± 0.21	0.84 ± 0.01	12.83 ± 0.11	3.23 ± 0.01
B-SM	0.11 ± 0.15	8.58 ± 0.10	0.44 ± 0.03	12.43 ± 0.11	3.21 ± 0.01
C-SM	0.17 ± 0.10	9.80 ± 1.12	0.64 ± 0.03	12.50 ± 0.34	3.19 ± 0.02
A-GR	1.09	6.01	0.27	14.9	3.20
B-GR	0.25	5.41	0.31	14.9	3.20
C-GR	0.17	7.10	0.42	14.9	3.13
A-CA	0.18	7.92	0.45	14.3	3.20
B-CA	0.45	8.13	0.44	13.9	3.18
C-CA	0.15	8.97	0.56	13.9	3.16


*Abbreviations as in **Table 2**. Standard deviations were indicated only when triplicates has been used (Synthetic Must fermentations).*

### Volatile Compound Production in Industrial Fermentations

The volatile profiles of the six final wines were evaluated (Supplementary Table S2). A total of twenty-nine volatile compounds were quantified and classified into esters (10), alcohols (8), acids (7), carbonyl compounds (3) and lactones (1). Among esters, the most abundant in all fermentations was ethyl acetate, followed by ethyl lactate. However, ethyl and isoamyl acetates, ethyl hexanoate and ethyl butyrate were detected above the odor threshold only in GR wines. In the case of alcohols, isoamyl alcohol, isobutanol and ß-phenylethanol were the main alcohols detected in all wines. All of them and methionol were present above the odor threshold, except for isobutanol in GR-C wine. Acetic acid was by far the most abundant acid in both wine varieties. All except isobutyric acid and decanoic acid were present above the odor threshold. Additionally, the major carbonyl compounds acetaldehyde and butyrolactone were present in all fermentations in a similar range, but the latter was detected below the odor threshold.

### Sensory Analysis of the Industrial Wines

The wines obtained at industrial scale for the three different treatments underwent sensory evaluation by triangle tests. **Table 4** presents the results obtained for the two different varieties. Statistically significant differences among the wines were found in three of the four tests performed, as more than 10 of 17 panelists were able to differentiate wines produced with different inocula. In the case of GR wines, native *S. cerevisiae* fermentations were different from the fermentation produced with the commercial strain of *S. cerevisiae*. When the CA variety was tasted, the wines presented in both sensory tests were perceived as different.

**Table 4:** Triangle test evaluation of final industrial wines.

Triangle test	Correct answers (Total)
A-GR against B-GR	7 (17)
B-GR against C-GR	12^∗∗^ (17)
A-CA against B-CA	12^∗∗^ (17)
B-CA against C-CA	10^∗^ (17)


*Abrreviations as in **Table 2**. ^∗∗^Significant difference *p*-value < 0.01. ^∗^Significant difference *p*-value < 0.05.*

## Discussion

In this work, the effects of native multi-starter yeast inocula on industrial and laboratory alcoholic fermentations have been studied. Concerning fermentation kinetics and total yeast population, similar results were obtained for the fermentations, and thus similar behavior was found between commercial and native yeast inocula. Additionally, the data obtained followed the typical growth pattern, with values of total yeasts at the end of the alcoholic fermentation close to 10^8^ CFU/mL. This value is consistent with results obtained from pure *S. cerevisiae* fermentations as well as from combined *S. cerevisiae* and non-*Saccharomyces* vinifications (Beltran et al., 2002; Gobbi et al., 2013; Belda et al., 2015). However, minor differences have been observed when natural and synthetic must were compared. Natural must was the best medium to grow the *Saccharomyces* yeast, because the recovery on YPD plates was more than 60% at mid fermentation, while in synthetic must the presence of *Saccharomyces* was reduced to 5%. However, *S. cerevisiae* was the only isolated at the end of all fermentations. Although synthetic must tries to mimic natural musts, these are more complex and most likely will be richer in nutrients, which could be a determining factor.

The low yeast population size, such as the observed in GR musts, is normally related to low recovery of the main non-*Saccharomyces* species (*H. uvarum* and *S. bacillaris*), which allows minor species to be easily detected (Beltran et al., 2002). Alternatively, CA must exhibits the typical Priorat microbial fingerprint consisting mainly of *H. uvarum* and *S. bacillaris* (Padilla et al., 2016). It is important to highlight that all non-*Saccharomyces* species isolated at this point have been previously reported on grapes or wine fermentations from the Priorat region (Torija et al., 2001) and are fairly universal, as reviewed by Jolly et al. (2014).

When comparing the populations obtained using culture-dependent and culture-independent techniques in fresh must samples, total yeast plate counts were approximately 1-log lower than qPCR data when yeast populations were low. The qPCR of the *Saccharomyces* spp. population in both varieties found approximately 10^3^ cells/mL, but no isolates from these species were recovered from the fresh juice. Similar qPCR results had been reported during the characterization of Merlot musts, but in that case, the culturing of *S. cerevisiae* was directly excluded due to the choice of a non-*Saccharomyces* growth media (Zott et al., 2010). However, the qPCR determination of *H. uvarum* population overestimated it at this initial point, as previously reported, and therefore our data support the suggestion that qPCR is a more sensitive method concerning detection of this species (Zott et al., 2010). In contrast, *S. bacillaris* was slightly underestimated. The reasons for these differences could be different: on one side the differential growth of different species on plates, and on the other side due to limited specificity of the qPCR probes and the method efficiency.

Yeast counts and population dynamics after the incorporation of native yeast were also monitored. The initial growth of the non-*Saccharomyces* yeasts was only observed clearly using synthetic must, while the use of natural must seems to be more restrictive to the growth of this type of yeasts. However, this fact is not so clear in all fermentations, because the increase of non-*Saccharomyces* populations by plating has been detected in the GR fermentations inoculated with commercial starter and CA fermentations inoculated with native starter, while this increase is no so evident in other fermentations. The overall detection and quantification of yeast during different fermentation strategies by both culture-dependent and independent methods were very similar, as also reported by Zott et al. (2010), likely due to the high yeast population levels and small number of dominant species. However, small differences need to be further described. One of the main differences is that the non-*Saccharomyces* yeasts were detected up to mid fermentation by plating but until the end of the fermentation by qPCR analysis, as previously reported (Hierro et al., 2006). In these fermentations, *Hanseniaspora* spp. values ranged from 3 × 10^4^ to 3 × 10^6^ cells/mL at the end of the different industrial fermentations, while *S. bacillaris* counts were approximately 3 × 10^5^ cells/mL in the final CA wines, which was in agreement with previous studies (Hierro et al., 2007; Andorrà et al., 2010; Zott et al., 2010). Additionally, *T. delbrueckii* was detected and quantified by qPCR in fermentations A-GR, A-CA and B-CA, but it was only isolated from experiment A-GR at 24 and 48 h after non-*Saccharomyces* inoculation. At these points, qPCR detected *T. delbrueckii* populations at 4 × 10^4^ and 1 × 10^5^ cells/mL, values above the cell concentrations found in CA fermentations. Disagreements in the detection of this species in plates and qPCR were also reported by Zott et al. (2010).

In addition to yeast identification, isolates from the main species were typified to assess the dominance of the starter culture. In experiments where a mixture of three native *S. cerevisiae* strains was inoculated, 100% of *S. cerevisiae* isolates exhibited the electrophoretic pattern of the inoculated strains. This result indicates that the three native *S. cerevisiae* strains included in the yeast consortium coexisted throughout the alcoholic fermentation and dominated the process, being in all types of fermentations (synthetic, Grenache and Carignan) a clear predominance of CECT13132, independently of the composition of the must or the presence of other native yeasts. Similarly, in a study conducted in Albariño white wines where three native strains were singly inoculated, all strains were recovered, and the percentage of imposition was between 90 and 100% in the different stages of fermentation (Carrascosa et al., 2012). In contrast, other authors (Esteve-Zarzoso et al., 2000; Sun et al., 2015) have reported that not all commercial yeast starters can dominate the fermentations in comparison with natural yeast present or isolated from their area, showing that the native microbiota prevailed over the commercial starter culture used, mainly isolated from other oenological region. This result supports the idea that autochthonous yeasts are well adapted to particular fermentation conditions, and thus their incorporation in a mixed inoculum is highly recommendable.

Wines produced with commercial strains rendered higher levels of glycerol and acetic acid, but in all cases, the final content was acceptable. These commercial strains are among the most used in the region and are able to perform the alcoholic fermentation of high sugar content to dryness. In the case of GR, all fermentations produced the same final alcohol content. However, for CA wines, there were some differences in the alcohol production. This result could be due to the heterogeneity of the starting must, which could include slight differences in the sugar content.

The volatile profiles of the different wines were also studied. The total acid content was higher in fermentations conducted using commercial *S. cerevisiae* strains, in agreement with results obtained in the general chemical characterization. However, the concentrations of the other analyzed volatile compounds were very similar among different treatments. The contributions of *H. uvarum*, *C. zemplinina*, *T. delbrueckii*, and *M. pulcherrima* to wine aroma have been studied (Comitini et al., 2011; Andorrà et al., 2012; González-Royo et al., 2014; Loira et al., 2014; Renault et al., 2015). Most studies concluded that the incorporation of these species exhibited a positive impact on aroma development. Nevertheless, most articles focused on evaluating the effects of single strains or mixed starters composed of one *S. cerevisiae* strain and one non-*Saccharomyces* species. Therefore, the interactions among different non-*Saccharomyces* wine yeast species need to be further elucidated. The results obtained in this paper highlight that complex interactions among yeast strains are likely to occur during the industrial fermentation of grape juice, and thus it is difficult to identify clear trends among different inoculation strategies. Still, the sensory evaluation concluded that most of the wines produced could be identified as different from the organoleptic point of view. However, high ethanol content and the full body characterize the Priorat wines, which is the consequence of its high complexity. Thus, although tasters could differentiate all the produced wines, there was not a significant preference: in all the cases the preferences were close to 50% of the tasters that identified the differences.

## Author Contributions

BP, LZ, AF, RP, and BE-Z performed the experiments; BP, BE-Z, GB, and AM designed the experiments, analyzed and interpreted the results; wrote the manuscript.

## Conflict of Interest Statement

The authors declare that the research was conducted in the absence of any commercial or financial relationships that could be construed as a potential conflict of interest.

## Footnotes

1 http://www.yeast-id.com
