# Supplementary material for: Sequential Inoculation of Native Non-Saccharomyces and Saccharomyces cerevisiae Strains for Wine Making
Source: Front Microbiol. 2017 Jul 18;8:1293. doi: 10.3389/fmicb.2017.01293 (PMC5513938; doi:10.3389/fmicb.2017.01293)
Supplement: Supplementary file 1 [file Table_1.DOCX]

Supplementary Table S1. Correlation coefficient (R2), slope and efficiency of standard curves obtained from serial dilutions of yeast cells. Efficiency was estimated by the formula E=((10^-1/slope^)-1)x100

| Target | R^2^ | Slope | Intercept | Efficiency (%) |
| --- | --- | --- | --- | --- |
| *Saccharomyces spp.* | 0,9943 | -3,6904 | 37,613 | 86,62651 |
| *Hanseniaspora spp.* | 0,9907 | -3,8253 | 40,991 | 82,56495 |
| *S. bacillaris* | 0,9961 | -3,0722 | 38,145 | 111,5922 |
| *T. delbrueckii* | 0,9823 | -3,3613 | 39,199 | 98,38277 |
| *M. pulcherrima* | 0,9927 | -2,7820 | 37,517 | 128,7987 |

Supplementary material Table 2. Concentration of volatile compounds in final wines (mg/L). A: Mixed fermentation( 4 species of non-*Saccharomyces* and the three strains of *Saccharomyces*), B: Fermentation performed using the three native strains of *Saccharomyces*, C: Fermentations conducted by industrial yeast starter belonging to Saccharomyces

|  | Odor thershold | Grenache | | | Carignan | | |
| --- | --- | --- | --- | --- | --- | --- | --- |
| Compound |  | A | B | C | A | B | C |
| **Esters** |  |  |  |  |  |  |  |
| Ethyl acetate | 12.3 | 51.43* | 42.58* | 60.82* | 47.32* | 59.15* | 42.42* |
| Isoamyl acetate | 0.03 | 0.86* | 1.4* | 1.68* | 0.63* | 0.58* | 0.88* |
| Hexyl acetate | 1.5 | <D.L. | <D.L. | 0.02 | <D.L. | <D.L. | <D.L. |
| Ethyl propanoate | 5.5 | 0.05 | <D.L. | <D.L. | <D.L. | 0.07 | 0.06 |
| Ethyl butyrate | 0.125 | 0.21* | 0.27* | 0.28* | 0.11 | 0.11 | 0.08 |
| Ethyl hexanoate | 0.062 | 0.32* | 0.6* | 0.39* | 0.24* | 0.26* | 0.28* |
| Ethyl octanoate | 0.58 | 0.1 | 0.15 | 0.1 | 0.1 | 0.1 | 0.13 |
| Ethyl decanoate | 0.2 | <D.L. | <D.L. | <D.L. | <D.L. | <D.L. | <D.L. |
| Ethyl lactate | 154 | 10.33 | 10.24 | 10.6 | 6.33 | 8.48 | 6.52 |
| Diethyl succinate | 200 | 0.27 | 0.25 | 0.41 | 0.29 | 0.28 | 0.23 |
| Total Esters |  | 63.57 | 55.49 | 74.3 | 55.02 | 69.03 | 50.6 |
| **Alcohols** |  |  |  |  |  |  |  |
| Isobutanol | 40 | 60.45* | 52.53* | 31.26 | 86.61* | 81.98* | 57.78* |
| 1-Butanol | 150 | 0.99 | 1.02 | 1.39 | 0.63 | 0.69 | 0.92 |
| Isoamyl alcohol | 30 | 246.41* | 232.85* | 244.32* | 292.87* | 280.07* | 357.22* |
| 1-Hexanol | 8 | 1.57 | 1.39 | 1.31 | 1.45 | 1.56 | 1.4 |
| *cis*-3-Hexenol | 0.4 | 0.15 | 0.16 | 0.1 | 0.1 | 0.1 | 0.09 |
| Methionol | 1 | 1.51* | 1.89* | 1.39* | 2.38* | 2.01* | 3.41* |
| Benzym alcohol | 200 | 0.23 | 0.01 | 0.28 | 0.11 | 0.09 | 0.09 |
| ß-Phenylethanol | 14 | 35.63* | 32.95* | 31.76* | 50.66* | 48.04* | 72.74* |
| Total Alcohols |  | 346.94 | 322.8 | 311.81 | 434.81 | 414.54 | 493.65 |
| **Acids** |  |  |  |  |  |  |  |
| Acetic acid | 300 | 366.32* | 362.48* | 454.47* | 367.23* | 320.18* | 487.19* |
| Butyric acid | 0.173 | 0.86* | 1.01* | 1.03* | 0.66* | 0.6* | 0.57* |
| Isobutyric acid | 2.3 | 1.53 | 1.5 | 0.77 | 1.88 | 1.56 | 1.5 |
| Isovaleric acid | 0.033 | 1.38* | 1.41* | 1.34* | 1.61* | 1.61* | 2.51* |
| Hexanoic acid | 0.42 | 3.03* | 3.75* | 2.7* | 1.83* | 1.5* | 1.43* |
| Octanoic acid | 0.5 | 3.22* | 4.56* | 2.68* | 1.88* | 1.42* | 1.36* |
| Decanoic acid | 1 | 0.36 | 0.51 | 0.31 | 0.32 | 0.54 | 0.35 |
| Total Acids |  | 376.7 | 375.22 | 463.3 | 375.41 | 327.41 | 494.91 |
| **Carbonyl compounds** |  |  |  |  |  |  |  |
| Acetaldehyde | 0.5 | 3.86* | 3.75* | 3.22* | 3.22* | 3.29* | 3.61* |
| Diacetyl | 0.1 | 1.49* | 1.94* | 1.04* | <D.L. | 0.5* | 0.86* |
| 3-Hydroxybutanone | 150 | 1.63 | 1.98 | 1.22 | 1.02 | 1.12 | 1.7 |
| Total Carbonyl compounds |  | 6.98 | 7.67 | 5.48 | 4.24 | 4.91 | 6.17 |
| **Lactones** |  |  |  |  |  |  |  |
| γ-Butyrolactone | 35 | 6.46 | 5.97 | 7.85 | 6.7 | 6.77 | 8.32 |

*: above odor threshold

<D.L.: below detection limit
